# Effect of Temperature-Dependent Bacterial Growth during Milk Protein Fractionation by Means of 0.1 µM Microfiltration on the Length of Possible Production Cycle Times

**DOI:** 10.3390/membranes10110326

**Published:** 2020-11-02

**Authors:** Simon Schiffer, Ulrich Kulozik

**Affiliations:** Chair of Food and Bioprocess Engineering, School of Life Sciences, Technical University of Munich, 85354 Freising, Germany; ulrich.kulozik@tum.de

**Keywords:** skim milk, microfiltration, production time, temperature

## Abstract

This study determined the maximum possible filtration time per filtration cycle and the cumulated number of operational hours per year as a function of the processing temperature during milk protein fractionation by 0.1 µm microfiltration (MF) of pasteurized skim milk. The main stopping criteria were the microbial count (max. 10^5^ cfu/mL) and the slope of the pH change as a function of filtration time. A membrane system in a feed and bleed configuration with partial recirculation of the retentate was installed, resembling an industrial plants’ operational mode. Filtration temperatures of 10, 14, 16, 20, and 55 °C were investigated to determine the flux, pH, and bacterial count. While the processing time was limited to 420 min at a 55 °C filtration temperature, it could exceed 1440 min at 10 °C. These data can help to minimize the use of cleaning agents or mixing phase losses by reducing the frequency of cleaning cycles, thus maximizing the active production time and reducing the environmental impact.

## 1. Introduction

The microfiltration (MF) of skim milk with a nominal pore size (nps) of 0.1 µm can be used to obtain two major milk protein fractions, whey protein (d = 4–8 nm) and casein (d = 40–400 nm). The purity of the whey protein fraction during MF depends, for instance, on the applied processing temperature [1,2]. Filtration temperatures between 50 and 55 °C are traditionally used because the low permeate viscosity at high temperatures results in a high flux or permeate mass flow. Furthermore, these filtration temperatures are often used to avoid β-casein migration from the micelle into the serum and to thus achieve high yields and purities of both fractions, i.e., caseins in the retentate and whey proteins in the permeate. At these temperatures, hydrophobic protein interactions are enhanced, resulting in a shift in the equilibrium between soluble casein monomers (mainly β-casein) in the serum and casein molecules integrated in the micelles [3], which can thus be retained by the membrane. It is, however, not considered that the growth of thermophilic microorganisms is enhanced at higher filtration temperatures [4].

Besides protein fractionation, studies and practical applications have shown that MF with large nominal pore sizes of 0.8–1.4 µm can be used to reduce the bacterial count in skim milk for producing consumption milk with an extended shelf life (ESL) without removing significant amounts of protein [5,6,7,8,9]. However, the 0.1 µm membranes used in the dairy industry for milk protein fractionation also retain most bacteria, which thus accumulate in the retentate. Notably, it is common in the industry to partially recirculate the retentate during MF or ultrafiltration, where, for example, higher counts of microorganisms accumulate in the feed flow with time during the crossflow MF before a diafiltration step [10,11]. These effects support biofouling on the membrane surfaces, which increases the fouling resistance and may become critical in terms of hygiene due to the fact that microbial growth can occur in deposited protein layers similar to biofilms produced by the microbiome itself. Long runs of filtration processes enrich the bacterial count in the retentate above the critical concentration of 10^5^ per mL according to statutory regulations applied in various countries. However, the critical concentration can differ depending on national laws or internal industrial quality regulations. This means that a colony-forming unit accumulation above this limit results in products that cannot legally be produced.

Although pasteurized skim milk is commonly used in industrial operations for milk protein fractionation to reduce the initial bacterial concentration, various thermostable microorganisms and spores can survive the pasteurization process [12]. Common thermostable microorganisms are, e.g., *Pseudomonadales* [12], as well as *Anoxybacillus flavithermus*, *Geobacillus* spp., and *Bacillus* spp., which can produce enzymes and acids [4], and thus influence the protein fractionation process via biofilm formation and pH reduction. The growth optima of these bacteria are between 40 and 65 °C [13]. Furthermore, enzymatic activity can occur after the pasteurization process due to thermostable enzymes, e.g., lipase and protease, produced by psychrotolerant bacteria prior to the pasteurization process [14]. Thermostable bacteria, such as *Pseudomonas* spp., can also produce proteolytic or lipolytic enzymes after the pasteurization process [15,16]. A filtration temperature of 55 °C, which is used in industrial applications, may therefore lead to an undesired proliferation of thermophilic microorganisms in the filtration system, with negative consequences on plant hygiene, pH development, and filtration performance. Furthermore, an attachment of bacteria on stainless steel [17] or in plant segments, which is generally addressed in various documents from the European Equipment and Design Group (EHEDG) proposing hygienic design principles [18], enables bacteria to reproduce inside of production units. This can become the deciding factor for the length of production time before another cleaning cycle is required to keep the plant hygiene under control.

Therefore, a trend toward lower processing temperatures can be observed [1,19,20]. Jarto et al. [19] have studied the effect of MF temperatures of 5 and 13 °C on the flux, initial flux decline, and separation efficiency in terms of the purity of the casein/whey protein fractions. Microbial growth, however, was not considered in this study. The downside of lower temperatures is that some psychrotolerant Gram-negative spore-forming bacteria can survive pasteurization [21], but the proliferation of most psychrotolerant bacteria is slow at low temperatures, such as 5 and 7 °C [22]. There are hardly any reports on the effect of temperature in the range above 10 °C on the microbial growth in dairy membrane operations regarding plant hygiene and filtration performance.

Furthermore, the energy demand for the process must be taken into account. If a decision has been taken for the low-temperature regime, from an environmental perspective, it then could be considered that a membrane plant should be operated at temperatures that are not as low as 10 °C to limit the energy expenditure to run the cooling systems required for temperature control. This is required to compensate for the energy introduced by the circulation pumps in the crossflow membrane plants. Therefore, there is an interest in operating MF plants at moderate temperatures between 10 and 20 °C. This, however, might conflict with increased microbial growth. 

Hence, there might be an option to find a balance between the criteria of flux, protein fractions’ purity, hygiene, and energy demand by selecting a filtration temperature above 10 °C. The limiting factor could be that all groups of bacteria, such as psychrotolerant, meso-, and thermophilic bacteria become increasingly active at temperatures between 10 and 20 °C, even if at a lower reproduction rate compared to their optimal growth temperatures [23]. In other words, a solution for this conflict of targets between plant and product hygiene and reduced energy consumption has to be identified.

Head-to-head studies comparing low and high filtration temperatures regarding microbial growth and MF performance criteria over filtration time have not yet been considered. Therefore, this study addressed a knowledge gap by assessing the effect of filtration temperature in the range of 10–20 °C and at 55 °C on the growth of microorganisms in the 0.1 µm MF systems used for milk protein fractionation. The purpose was to identify at which filtration temperature the hygiene conditions could be kept under control in the range of 10 to 20 °C, and therefore, to define how the production time between two cleaning cycles could be maximized. Since both microbial growth and the required energy for the cooling of a large-scale industrial system are responsive to changes in temperature, it is critical to assess the effect of temperature using small temperature increments. From the data obtained, the possible production cycle times at these temperatures were assessed using the 10^5^ cfu/mL and the pH development over time as decision criteria.

## 2. Materials and Methods

### 2.1. Skim Milk and Microfiltration Plant

Pasteurized skim milk (74 °C for 28 s) was obtained from a local dairy (Molkerei Weihenstephan GmbH & Co. KG, Freising, Germany) and stored at 4 °C until use. For each filtration experiment, a new polyethersulphone (PES) membrane cassette with a nominal pore size of 0.1 µm and an active membrane surface area of 0.093 m^2^ (Pall, Port Washington, WI, USA) was used. This membrane provides a large membrane area relative to the hold-up volume. In addition, it is a flat sheet membrane with the layers separated by spacer sheets similar to that of industrial spiral-wound membrane units. Furthermore, this membrane provides a high enough membrane area to remove the required amounts of permeate from the given volume of milk to be filtered. The membranes were selected based on supplier specifications regarding water flux to avoid the effects of different initial permeation rates.

**Figure 1:**
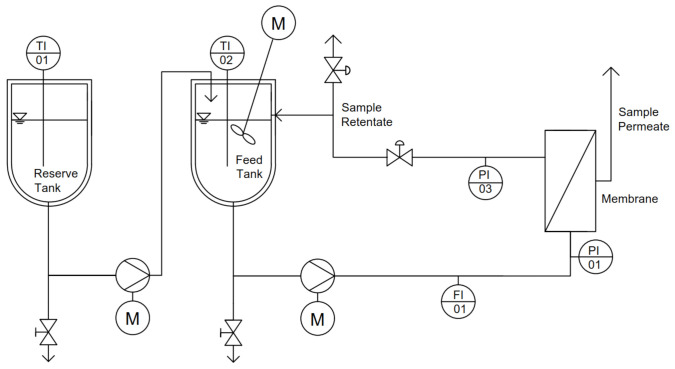
Piping and instrumentation scheme of the filtration plant (TI: temperature indicator; FI: flow indicator; PI: pressure indicator; M: motor).

The experiments were performed with a filtration setup, as schematically shown in Figure 1. During the experiments, a double-jacketed feed tank (V = 5 L) was used to temper the skim milk to the target temperature. A VGS430 gear pump (Verder Deutschland GmbH & Co. KG, Haan, Germany) generated a constant volume flow and a transmembrane pressure of Δp_TM_ = 1.1 bar (p_1_ = 1.2, p_2_ = 1.0 bar) at 55 °C and 20 °C, Δp_TM_ = 1.2 bar (p_1_ = 1.4 bar, p_2_ = 1.0 bar) at 16 °C and 14 °C, as well as Δp_TM_ = 1.3 bar (p_1_ = 1.6 bar, p_2_ = 1.0 bar) at 10 °C. The permeate and 10 L h^−1^ of the retentate were continuously removed during the filtration process and replaced with skim milk to keep the filling level of the feed tank the same over time. The remaining retentate was recirculated into the feed tank to obtain conditions comparable to industrial feed and bleed systems. To avoid temperature gradients, the feed tank was ideally mixed using a stirring system.

### 2.2. Microfiltration of Skim Milk

Before the experiment, the filtration plant and external feed tank were disinfected with 1% Hydrosan Stabil (Wigol, Worms, Germany) for 1 h at 30 °C and flushed with softened water. Then, the membrane was installed, caustic conditioning with 0.5% Microl Mix MAT Flüssig Plus (Wigol, Worms, Germany) was conducted for 20 min at 55 °C, and the system was flushed with softened water. During the conditioning and cleaning (Section 2.3), the pressures p_1_ = 0.4 bar, p_2_ = 0.2 bar, and Δp_TM_ = 0.3 bar were applied. The duration of the filtration experiments was dependent on the colony-forming unit growth and the development of the pH.

Furthermore, the pH in the feed tank of the product was monitored by a Protamess 911 pH-meter (Knick Elektronische Messgeräte GmbH & Co. KG, Berlin, Germany). The dry matter of the retentate was kept constant at 10 ± 0.2% and therefore continuously controlled using a Smart 6 system (CEM Cooperation, Matthew, NC, USA) to avoid a change in the feed composition.

### 2.3. Cleaning

After the filtration experiment, the filtration plant was flushed twice with softened water and caustic cleaning was applied with 1.5% Microl Mix MAT Flüssig Plus (Wigol, Worms, Germany) at 55 °C for 40 min. Additionally, the plant was flushed twice with softened water, and acidic cleaning with 0.5% nitric acid (60%) (Halag Chemie AG, Aadorf, Switzerland) was conducted at 55 °C for 20 min and rinsed twice with softened water.

### 2.4. Total Bacteria Count

A sample of the pasteurized skim milk was collected before each filtration. Furthermore, the retentate samples were collected every 60 min directly from the retentate outlet to avoid cross-contamination and stored at 4 °C until analysis. The colony-forming units were determined using a serial dilution in duplicate in sterile Ringer´s solutions and plated on a nutrient ager. The samples were incubated at 37 °C for 48 h. Plates with up to 300 cfu were used according to the established microbiological standard method [24] to determine the bacteria count. In cases where the colony-forming units per plate exceeded 300, only the higher dilution steps were used for the calculation of the microbial count. For details of the analytical method, see Marx et al. [25].

### 2.5. Data Evaluation and Statistical Analysis

The data were plotted using OriginPro 2017G (OriginLab Corporation, Northampton, MA, USA). The filtration experiments were performed in single trials with short measurement intervals of 30 min for pH and flux determination over a total filtration time of 600–1440 min. Error bars for the total bacteria count represent the max./min. values of two repetitions per sample.

## 3. Results

In the following, the flux, the microbial count, and the pH were measured as assessment criteria as a function of the filtration temperature and the filtration time. Finally, the resulting possible filtration cycle times and the related annual production (i.e., filtration) times were calculated based on these data.

### 3.1. Impact of Temperature on the Filtration Performance over Time

**Figure 2:**
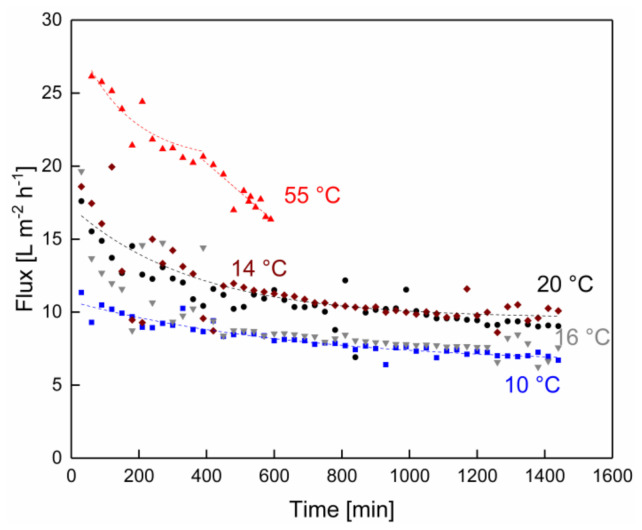
Flux as a function of the filtration time at processing temperatures of 10 (■), 14 (◆), 16 (▼), 20 (●), and 55 °C (▲). Dotted lines are guides for the eye.

As shown in Figure 2, the flux declined steeply from an initial value as a function of filtration time. This decline was more pronounced at 55 °C. This initial flux decrease was an effect of the adsorption and deposit layer formation by the retained protein fraction on the membrane surface, as shown by Rezaei et al. [26]. At first sight, the flux decline at 55 °C seemed to follow a linear trend. A closer assessment of the flux over time at 55 °C, however, showed that the initial flux decline tended toward an asymptotical shape up to 420 min, although this trend was not very obvious. It then seemed to decline against this trend again more steeply in the range of 420–600 min of filtration time, when the filtration was deliberately stopped due to reasons related to a sudden drop in pH, which occurred in the same time frame. A flux decrease as a function of an increasing protein concentration in the retentate flow during the microfiltration of skim milk, as described by Zulewska and Barbano [27], could cause a flux decrease over a long filtration duration. Since the dry matter level was kept constant by the feed-and-bleed mode, it can be excluded that an accumulation of dry matter in the feed caused these changes in flux.

The flux for the lower temperatures declined steeply at first, too, but then continued to decline at a lower rate up to 24 h, asymptotically reaching almost steady-state levels. The initial flux decrease during the microfiltration of skim milk was in accordance with the findings reported by Jarto et al. [19] for a skim milk microfiltration filtration at 13 °C. Measurements in this study could have been continued in these experiments but had to be stopped due to organizational reasons. The lowest flux level over time was obtained for 10 °C. The flux levels over time in the low-temperature range were relatively close together due to the small related changes in permeate viscosity. They roughly followed the order of the temperatures but only small differences were measured between 14 and 16 °C, probably due to the small temperature difference and minor confounded effects in the experimental procedure related to membrane properties or milk composition.

A related study at the temperature level of 50 °C was reported by Hartinger et al. [1], who also observed a steady and steep flux decline in milk MF up to 500 min of filtration time using spiral-wound membranes. However, in their study, the pH and colony-forming unit levels over time were not measured. These authors explained their observations in terms of progressing casein–whey–protein interactions at 50 °C and long contact times, which are further favored by the high protein concentration inside the deposited protein layer. Over time, this results in a layer with a higher filtration resistance with decreasing porosity and/or a greater height. These explanations are also supported by reports of other authors, e.g., Iametti et al. [28], Ng et al. [29], and Steinhauer et al. [30].

### 3.2. Changes in pH and Microbial Count as a Function of the Filtration Time

**Figure 3:**
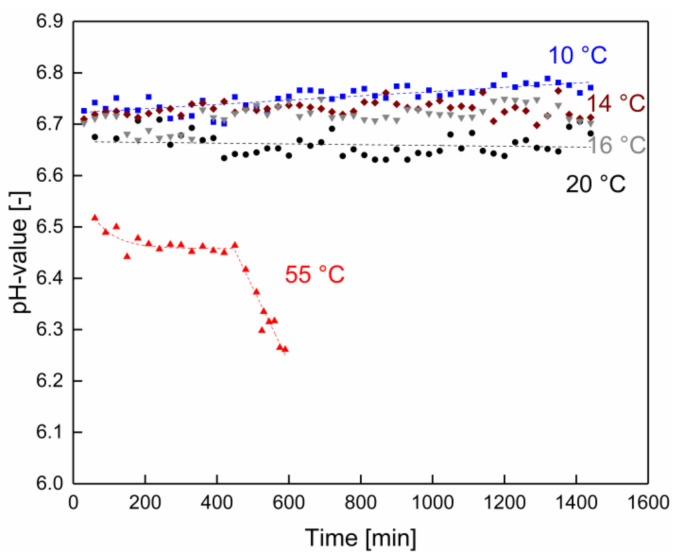
pH value as a function of the filtration time at processing temperatures of 10 (■), 14 (◆), 16 (▼), 20 (●), and 55 °C (▲). Dotted lines are guides for the eye.

The open question concerned how the more rapid flux decline beyond approximately 420 min filtration time at 55 °C could be explained. Figure 3 clearly shows that the pH remained stable throughout the entire duration of the experiment at the native milk pH for the experiments in the lower temperature range. There was only a slightly decreasing trend for the pH with increasing filtration temperature from 6.74 at 10 °C to 6.65 at 20 °C. In contrast to this, at 55 °C, the initial pH had already decreased by 0.4 pH units but then remained relatively stable at least up to 420–450 min. The lower base pH at 55 °C can be explained by a progressing change in the ionic equilibrium at higher temperatures in milk [31]. However, then a very obvious, steep drop in pH was observed, limiting the run time in this case to about 7 h, which was in accordance with experiences using industrial MF installations. Even though the pH was still above pH 6.2, this drop in pH led to a denser, more compact deposit layer due to less repulsion between the deposited proteins and, therefore, more intense protein–protein interactions between the deposited casein micelles, as reported by Kühnl et al. [32]. Furthermore, for the MF of milk, a drop in pH can affect the proteins immobilized in the deposited layer at very high concentrations for many hours more than is observed in milk with low protein concentrations flowing through the MF plant in short times.

**Figure 4:**
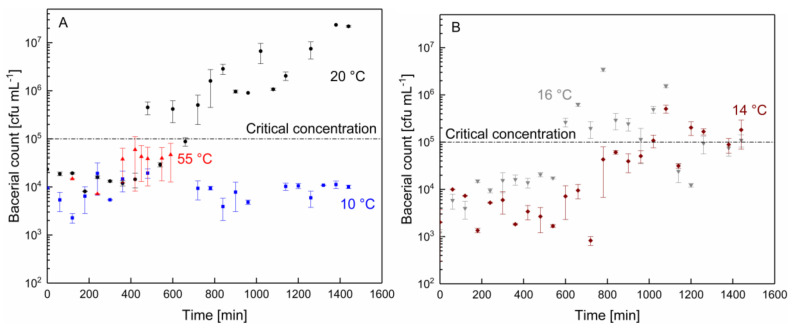
Bacterial count as a function of the processing time and temperature of (**A**) 10 C (■), 20 C (●), and 55 °C (▲), and (**B**) 14 °C(◆) and 16 °C (▼). Dotted lines are guides for the eye.

We further determined the microbial growth as a function of the filtration temperature. For the sake of clarity, the results are presented in separate diagrams in Figure 4A (55, 20, and 10 °C) and Figure 4B (16 and 14 °C). The critical concentration of 10^5^ cfu mL^−1^ can be altered depending on the applied national law or internal quality standards of the producer.

As can be seen, the microbial growth at 55 °C increased by almost one order of magnitude before the experiment was deliberately stopped because of the sudden drop in pH resulting from microbial activity, as shown in Figure 3. To explain this observation, a stronger biofilm formation by the thermophilic bacteria can be considered responsible for the steep drop in pH and strong flux decrease. The ability of common thermophilic microorganisms to form a biofilm and to multiply in this biofilm is known from studies reported by Chamberland et al. [4] and Sadiq et al. [33]. The activity of proteolytic enzymes was also considered as a possible factor for the rapid pH decrease at 55 °C since enzymatic protein hydrolysis leads to a reduced pH. However, Zhang et al. [34] showed an autolytic temperature at 55 °C for the AprX protease produced by *Pseudomonas*. Furthermore, Kroll and Klostermeyer [35] also reported an increased inactivation of proteinase produced by *Pseudomonas fluorescens* at 55 °C. Therefore, an increased protease activity did not seem to be the main effect with regard to the sudden drop in pH observed at 55 °C. 

At 10 °C, there was almost no increase in microbial count throughout the entire filtration time. At 14, 16, and 20 °C, the growth curves initially remained at the starting level for some time and then increased more steeply the higher the temperature became, finally reaching or crossing the critical colony-forming unit level of 10^5^ cfu mL^−1^ at 480 min for 20 °C (Figure 4A), 600 min for 16 °C, and 1020 min for 14 °C (Figure 4B). However, this increase in the colony-forming unit count in the retentate did not go along with a noticeable decrease in pH. In addition to thermophilic microorganisms, the spores of Gram-positive psychrotolerant microorganisms can also survive pasteurization [21]. During the filtration process, the remaining bacteria can settle and multiply at the membrane surface, where optimal nutritional conditions exist and spores can germinate within the deposits [36]. Biofilm formation on spiral-wound membranes is possible during the filtration of pasteurized milk at processing temperatures below 20 °C, as was shown by Chamberland et al. [37]. Furthermore, *Bacilli*, as well as *γ-Proteobacteria*, were observed on membranes in their study. Therefore, the bacterial growth between 10 and 20 °C was assumed to be due to the remaining psychrotolerant bacteria surviving the pasteurization process. Since the growth optima of most psychrotolerant bacteria are in the range of 20–30 °C [21], the time until a critical concentration of 10^5^ cfu mL^−1^ was exceeded was prolonged at lower temperatures. Furthermore, it has been shown that some meso- and thermophilic organisms can also grow at 15 °C [23], and therefore contribute to the bacterial count with increasing temperatures from 10–20 °C. Therefore, the critical colony-forming unit limit was reached at 480 min for 20 °C and at 1020 min for 14 °C. 

In summary and across all data presented so far, the conclusion is that, depending on the filtration temperature, different parameters determine the achievable filtration time. While at 55 °C, the microbial growth alone would allow for a longer filtration time, the sudden drop in pH led to a deliberate stop of the experiment after 10 h. At 20 °C, the pH remained constant, while the critical bacterial count was exceeded. From this perspective, the experiment could also have been stopped earlier after around 8 h if the colony-forming unit result had been available in time. Despite strong microbial proliferation, the stable pH was possibly due to the different metabolites formed by the bacteria growing at the different temperatures, although this question was outside the scope of this study and could be a subject of further investigations.

### 3.3. Calculation of the Possible Number of Production Cycles and Overall Annual Process Time

The obtained data regarding the length of the individual filtration cycle times were used for predicting the possible annual production time as a function of the filtration temperature. This information is useful for minimizing the number of cleaning-in-place (CIP) cycles, and thus to lower the loss in production time, loss of product and water into the unavoidable mixing phases, and the consumption of cleaning agents. It should be considered, however, that additional factors play a specific role in individual production scenarios. Therefore, the exact production time is obviously dependent on the filtration plant configuration and on-site processing conditions. Specifically, the age of the membrane and the associated irreversible long-term membrane fouling will have an impact. Furthermore, the bacterial composition of the raw material, which can vary over the year [38], can influence the possible production time. 

Regarding the bacterial count (with a critical concentration of 10^5^ cfu mL^−1^ in this study), flux stability, and pH at filtration temperatures of 10–55 °C, a certain production time was possible under the applied conditions (Table 1). For the following calculations for 10 °C, a production time of 1440 min was used, even though a distinct time over 24 h can be expected.

A cleaning cycle is required to recover the flux and remove irreversible fouling after each filtration run. The CIP-related cleaning time can vary depending on the application but can be set to 4 h according to realistic scenarios at the industrial level for MF units used for skim milk filtration. Therefore, considering the observed production time (t_filtration_) at a certain temperature and the time per cleaning cycle (t_cleaning_), the production time per year (t_production,year_) can be calculated using Equation (1):

(1) $$t_{production,\;year}=\frac{8760\;\frac{h}{a}}{\left(t_{filtration}+\;t_{cleaning}\right)}\;\cdot t_{filtration}$$

**Figure 5:**
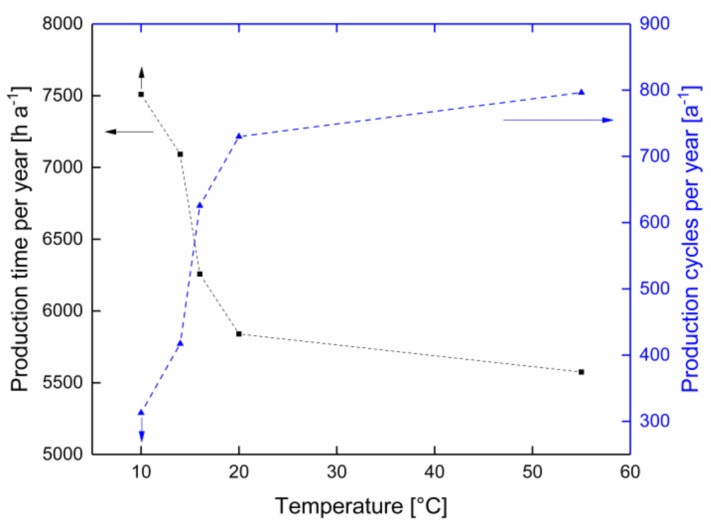
Production time (■) and the number of production cycles (▲) as functions of the product temperature (ϑ = 10–20 °C and 55 °C). Dotted lines are guides for the eye.

As shown in Figure 5, the possible annual production time increased with decreasing temperature from 5574 h a^−1^ at 55 °C to 7508 h a^−1^ at 10 °C. In conclusion, the data obtained in this study indicated that even if the production time per filtration could be prolonged at 10 °C for more than 24 h, it would not be possible to overcome the disadvantage of the reduced flux at lower filtration temperatures.

## 4. Conclusions

In this study, the effect of temperature (10–20 °C and 55 °C) on flux, pH, and microbial growth, and hence on the maximum filtration time was investigated. It was found that the limitation of the maximal possible production time during the MF of skim milk stemmed from different effects, depending on the filtration temperature. The number of annual production cycles was shown to decrease with temperature, and consequently, the number of cleaning cycles also decreased. This means that with each changeover from milk to water during shut-down and start-up of the MF plant [39], and within each CIP cycle, each change-over from water to lye, from lye to water, and so on, the environmental effect increases in terms of the water footprint and the consumption of cleaning agents. At 55 °C, approx. 800 cycles, and at 10 °C, a maximum of approx. 310 cycles can, or rather have to be, conducted annually, provided full-plant utilization across the year. This leads to more than twice the number of cleaning steps and four times the amount of product loss due to the mixing phase during the start and end of a filtration process, plus the mixing phases within each CIP operation. Therefore, when deciding which filtration temperature should be applied during the milk protein fractionation, the total filtration time over one year, the amount of cleaning agent, the product loss, and the energy consumption should be considered. Furthermore, the production at lower temperatures ensures a higher product quality regarding the bacterial count in the retentate. In the temperature range from 20–14 °C, an increase in the bacterial count up to the acceptable limit was observed after 400–600 min of production time (Figure 4). An increased bacterial count can affect the product quality. Therefore, considering all these aspects, filtration at 10 °C, possibly 12 °C, appeared favorable to ensure a constantly low bacterial count over a filtration time of at least 24 h. Finally, it should be noted that some conditions and also the decision criteria depend on the local situation, including the microbial raw milk quality, plant set-up, pasteurization conditions, and the critical limit chosen as the condition for ending a filtration cycle. In industrial plants, spiral-wound membranes are commonly applied. Due to slightly different flow conditions, the bacterial composition can be altered compared to the flat-sheet membranes used in this study with, however, similar spacer nets between the membrane sheets. A comparison between the two membrane systems at an industrial level is proposed. The results of this study can be used as a methodological guideline for the data-based assessment of the efficiency of the membrane plants in the dairy industry, particularly MF plants that are operated for milk protein fractionation, part of which could also be the quantification of the reduction of chemicals, as well as milk and water losses, in the mixing phases.

## Figures and Tables

**Table 1:** Possible production time as a function of temperature (ϑ = 10–55 °C) with limits set by the colony-forming unit count or pH.

Production temperature (°C)	10	14	16	20	55
Possible production time (h)	>24	17	10	8	7
